# Accounting for complexity in healthcare innovation debates: Professional views on the use of new IVF treatments

**DOI:** 10.1177/13634593221074874

**Published:** 2022-02-01

**Authors:** Alina Geampana, Manuela Perrotta

**Affiliations:** Aston University, UK; Queen Mary University of London, UK

**Keywords:** add-ons, clinical trials, commercialisation, evidence, IVF

## Abstract

Social scientists have long been interested in the forces and values driving healthcare innovation. The simultaneous rise of 20th century healthcare reforms, increased importance of evidence and upsurge in lay health activism have shaped modern medicine. On this backdrop, fertility care emerged in the 1970s. Recent developments reveal a contentious relationship between new fertility treatments and clinical evidence, with emerging technologies being used without conclusive evidence of effectiveness despite being sold to patients. Initial critiques of this phenomenon emphasise commercial interests as the culprit, suggesting that the problematic use of unproven treatments is mainly driven by the private sector. Here, we challenge this over-simplified view of IVF care. Drawing on a qualitative analysis of key documents and 43 in-depth professional interviews, this article identifies three main stakeholder approaches to new treatment adoption. We argue that viewpoints are anchored within three critical overarching ‘modes of coordination’ or core values in modern healthcare: efficiency, effectiveness and patient-centeredness. This analysis encourages a more contextualised overview of fertility care than previous literatures have afforded. The IVF case shows that an emphasis on private versus public clinic practices obscure similarities between the two along with the values motivating healthcare professionals’ approaches to new treatments.

## Introduction

The relation between medical knowledge production and the use of new treatments and technologies has been a core topic of investigation in sociological work (Moreira, 2012; Rabeharisoa and Bourret, 2009). Despite progress on innovation, medical uncertainty has been further engendered rather than eliminated (Callon et al., 2009). Public controversies such as those on alternative medicine, genetic screening and stem-cell research have increased the visibility of uncertainty and conflicting value systems. Uncertainty in fertility care, however, has received less attention from scholars.

Having only developed over the past four decades, fertility care has been characterised by rapid technological advancements (Hurley, 2013). Not unlike other areas of medicine, many of the treatments introduced and widely used now in clinical practice have not been scrutinised through evidence-based-medicine (EBM) protocols (Human Fertilisation and Embryology Authority [HFEA], 2020). The intensely-competitive UK fertility sector, where over 60% of treatments are funded privately, has been heavily criticised for making ‘false claims of effectiveness to take financial advantage of desperate couples’ (Rutherford, 2017: 1850). For professionals in agreement with this view, new treatments (commonly referred to as ‘add-ons’) remain contentious (Harper et al., 2017; Repping, 2019; Wilkinson et al., 2019). This view has shaped a ‘public versus private’ discursive division in professional discussions, where private greed is seen as the culprit behind the problematic adoption of new treatments. Drawing on data from in-depth interviews with fertility professionals, we argue that conflict in the add-on debate can be better understood through contextualising different professional views within modern organisational healthcare values or ‘modes of coordination’ (Moreira, 2012). In our analysis, we show how different, and sometimes competing, values underlie professionals’ views on IVF innovations. Interpretations of add-ons lie on a wider spectrum of opinions than has been presented in the media and the medical literature. For example, our research shows that both private and public practitioners use new treatments, thus complicating the perceived private/public divide. More broadly, we stress that the overuse of the public/private dichotomy in debates and the excessive focus on commercialisation obscures varied professional values on EBM and new treatment use. We contribute to the IVF literature by bringing together different issues previously discussed in the literature (e.g. the importance of patient experiences, commercial aspects of fertility care) and relating them to current debates on evidence and innovation – topics that have been discussed less in the IVF context. To set up the context for our findings, we first explain the details of the professional controversy. We then discuss the social science literature in a way that contextualises the add-on debate within larger fertility and healthcare practices. The methodology section outlines our sample and reasons for choosing Moreira’s (2012) modes of coordination framework for our analysis. The discussion section emphasises our reframing of this innovation debate and the article’s contributions to the sociological IVF and EBM literatures.

## The contours of the medical add-on debate

The add-on debate is a discussion that was prompted by a media storm, but that moved and remained in the professional realm subsequently. The start of the controversy is worth mentioning in as much as it draws the broad initial contours of the discussion. In November 2016, the IVF sector found itself in crisis mode: a BBC Panorama documentary entitled ‘Inside Britain’s Fertility Business’ warned against unproven additional treatments offered to fertility patients. It was private clinics in particular that came under fire following the investigation that highlighted the upselling of add-on treatments lacking randomised control trial (RCT) evidence that they increase live birth rates (LBRs). The Panorama discussion was underpinned by research conducted at Oxford University’s Centre for Evidence-Based-Medicine and the resulting publications (Heneghan et al., 2016; Spencer et al., 2016) stressed a lack of effectiveness evidence for IVF add-ons sold in private clinics. Critics called for the Human Fertility and Embryology Authority (HFEA) to take action to protect patients. This controversy was met with calls for a stricter EBM approach in IVF (Harper et al., 2017; Repping, 2019; Wilkinson et al., 2019). Such publications highlighted the need to conduct RCTs before offering new treatments. As such, the issue was defined in relation to: (1) the unacceptability of providing treatments without an evidence base and (2) protecting patients from such unproven treatments. However, as we detail further in our analysis, this framing failed to take into account the fact that many previous IVF treatments had been incorporated into clinical practice without a clear evidence base. It also failed to account for patient agency and choice, assuming that all IVF patients are gullible and in need of regulatory protection. Our analysis also details how, in practice, add-on choices are far more complex for stakeholders.

Regarding policy action on this issue, responsibility falls on the HFEA to take action. However, within the commercialised context of UK IVF, their purview is limited. While it cannot regulate treatment prices, the HFEA aimed to inform the public by providing online information on the effectiveness of the nine widely-used add-on treatments (popular examples include elective freeze-all cycles, embryo glue, endometrial scratching, preimplantation genetic screening (PGS) and time-lapse imaging). These are categorised according to a traffic light system informed by a strict EBM perspective. Treatments with no evidence of effectiveness are marked as red; those with limited or conflicting evidence are marked amber; and those with more than one high-quality RCT are meant to be green, although none have this classification currently. We analyse the HFEA’s policy decisions in more detail in our findings in order to relate their stance to the different values it exposes.

## Contextualising fertility care

The add-on debate raises questions regarding the role of commercialisation in IVF and whether it is driving the adoption of ineffective technologies. The influence of commercial forces in healthcare has been explored extensively in the social science literature (Conrad, 2005; Greene, 2007; Light, 2010; Timmermans and Oh, 2010). Modern shifts have rendered healthcare increasingly rationalised and driven by economic calculation (Light, 2000; Moreira, 2007). The global fertility care sector itself is expected to reach a value of over 37 billion USD by 2027 (Grand View Research, 2020). Fertility clinics have proliferated worldwide and, depending on the national care context, can be public, private, or both. In the UK, roughly 40% of IVF cycles are state-funded, while the rest are paid for out-of-pocket (HFEA, 2020). However, this does not necessarily mean all publicly-funded cycles are provided by a National Health Service (NHS) clinic. Some private clinics can provide treatment to state-funded patients through outsourcing, while some patients pay out-of-pocket at ‘public’ (NHS) clinics now increasingly operating under public/private partnerships (HFEA, 2020). The sociological IVF literature has been more focussed on the commercialised exchanges and political economies surrounding fertility care (Nahman, 2016; Pavone and Arias, 2012; Thompson, 2016; Van de Wiel, 2019; Vertommen, 2017), with less attention paid to innovation trajectories. The current stagnation of IVF success rates has led to intense competition for the development of new and promising biotechnologies (Macklon et al., 2019). However, the relationship between EBM and the adoption of new IVF treatments has never been a straightforward one.

In the UK, the emergence of evidence-based-medicine (EBM) played an important role in the restructuration of the healthcare system from the mid-1990s onwards (Ghali and Sargious, 2002; Harrison, 1998). The establishment of the National Institute for Clinical Excellence (NICE) was a direct result of this knowledge shift (Moreira, 2007). Working within the NHS, NICE provides trusts, clinicians and patients with clinical guidelines and advice on ‘best practice’ (Rawlins, 1999). Fertility care developed in the late 1970s and 1980s, before EBM took hold. Many common-practice IVF treatments used now, including the widely-used ICSI (intracytoplasmic sperm injection), were introduced without the support of clinical trials, even post-1990s. Nonetheless, the relationship between EBM and fertility care has been underexplored by social scientists, despite insightful analyses in other contexts, such as alternative medicine (Morreim, 2003), cancer treatment (Broom and Tovey, 2007) and paediatrics (Timmermans and Angell, 2001).

More attention has been paid to infertility patients’ experiences (Greil et al., 2009) and patient activism (Gunnarsson Payne and Korolczuk, 2016; Novotny and De Hertogh, 2019). Patient-centred medicine is also important to IVF professionals (Streisfield et al., 2015). This is part of a bigger trend in healthcare, whereby we have seen increased patient activism and lay challenges to the medical profession (Timmermans and Oh, 2010). Patients now are better able to make independent decisions regarding their care as a result of the increased democratisation of medical knowledge (Light, 2010). Self-management and autonomy have given rise to support communities as well as infertility patient advocacy groups (Speier, 2011). Overall, scholars (Epstein, 2008; Timmermans and Oh, 2010) note that the past decades have seen expanded interest towards the inclusion of patient voices in medical decision-making.

## Methodology

**Figure 1.**
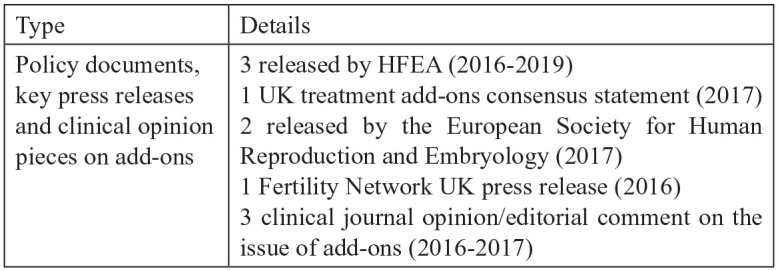
Document sample.

The data analysed in this article are part of a larger project investigating the use of new imaging technologies in IVF. Such technologies (commonly referred to as ‘time-lapse’) are categorised by the HFEA as add-ons. Consequently, our data collection had a significant focus on professional views on evidence for innovations. In this article, we draw on stakeholder interviews and relevant communications, articles and policy documents. The key documents have been chosen on the basis of their relevance to professionals and policy – many being cited in professional discussions. Please see Figure 1 below for details on the document sample.

**Figure 2.**
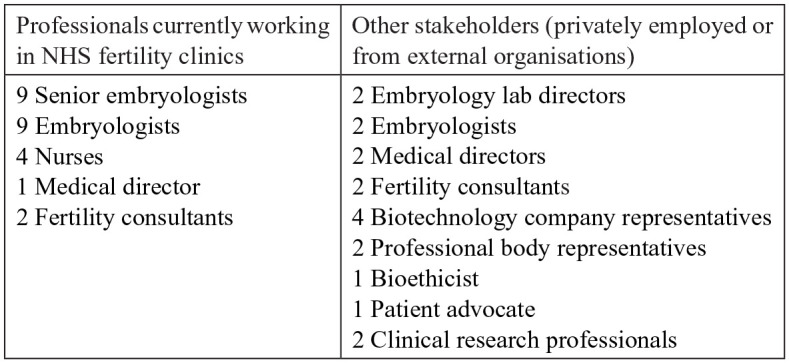
Respondent sample (total: 43 interviewees).

We also include data from 43 stakeholder interviews (see Figure 2 below for details). Interviews were conducted by the authors between June 2017 and July 2019. Respondents working in NHS-affiliated clinics were recruited from five sites who agreed to participate in our larger study. In addition to university ethics approval, we received ethics clearance from the relevant NHS trusts and each clinic site separately. We selected clinics based on their use of add-ons and availability. All are located in England. Further details about location cannot be disclosed due to confidentiality concerns. The data collected are not publicly available due to privacy and ethical restrictions. An anonymised version will become available at a later date by request from our funder, following completion of the research project. We conducted a total of 25 interviews with NHS-affiliated staff, including fertility consultants, embryologists and nurses. The other interviewees (18) were recruited via email based on their expertise and included clinicians, researchers and representatives of professional bodies from England, Scotland and Wales. Due to the HFEA being the regulatory body for all of the UK and add-ons being offered across nations with similar concerns around private care, we stress the issues discussed in this article as relevant to the UK as a whole, with all countries having a relatively high percentage (40% in Scotland, 60% in England and Wales and 75% in Northern Ireland) of private clinic cycles (HFEA, 2020).

The majority of interviews (35) were conducted face-to-face, while the rest (8) were conducted on Skype or over the phone. All interviewees signed a consent form prior to their interview. Conversations lasted between 35 and 90 minutes and were audio-recorded and professionally transcribed. The interview guide was designed to obtain participants’ experiences with and opinions on add-ons, practice challenges and evidence issues in IVF treatment. The quotations used in the article were chosen based on their ability to: (1) concisely illustrate the themes presented; (2) showcase a variety of professional perspectives from respondents working in different roles. A methodological limitation is the lack of data on patient consultations. Due to feasibility and ethical concerns, we were not able to observe clinical consultations. Thus, we cannot comment on how information is disseminated to patients. Secondly, although we had wished to interview more regulatory professionals, we experienced a lower response in this group. To mitigate this, we ensured all relevant regulatory policy documents were included in our data.

Following data collection, we entered all documents and interview data into NVivo, a software package used for the analysis of qualitative data. The data were coded deductively by looking for themes from previous literature or discussed in advance. We also coded inductively where we identified new codes. New codes and larger themes were discussed between authors to reach agreement. The analysis process was continual and open-ended. We paid close attention to discussions of new treatments, the add-on controversy, evidence and its importance, as well as the use of strict EBM protocols in IVF. To guide data collection and subsequent analysis we aimed to discern between current priorities in fertility care and their relationship to the evidence controversy. However, we also paid attention to the social context and the positionality of the respondents in order to reflect on how this might influence their views (Wodak and Meyer, 2015). The analytical mode behind our analysis is best encapsulated by abductive analysis, where the researchers combine careful data analysis with a broad theoretical base (Timmermans and Tavory, 2012). Through the process of abductive analysis (Timmermans and Tavory, 2012), we identified three over-arching approaches (centred respectively on EBM, innovation or patient perspectives) to the add-on/evidence debate. These can be traced to current Western ideals in medicine, as we have outlined them in our literature review. As our analysis unfolded, we decided that Moreira’s (2012) mode of coordination framework can provide the best contextual understanding of the implications of our analysis as it provides a link between stakeholder values and larger shifts in healthcare, thus helping us understanding the debate beyond the private/public dichotomy.

### Framework of analysis

We use Moreira’s (2012) concept of ‘modes of coordination’ to account for how contrasting views emerged and are sustained in the evidence debate. Building on Boltanski and Thévenot’s (2006) work, he suggests that different modes of coordination in healthcare are supported by diverse orders of worth or systematic principles of evaluation. According to Moreira’s (2012), the development of modern Western healthcare has been characterised by three modes of coordination: 1. efficiency, supported by the ideal of ‘the market’, values productivity, financial benefit and consumer choice; 2. effectiveness, supported by the ideal of ‘the laboratory’, puts value on standardisation, evidence-based protocols and knowledge production; and 3. patient involvement, supported by the ideal of ‘the forum’, values lay participation in healthcare decision-making and social consensus. Each mode enables ‘articulation between different ways of knowing and moral conceptions of the role of healthcare in society’ (Moreira, 2012: 1).

## Findings

Our findings reveal that the evidence debate in fertility care has nuance and unresolved dilemmas. We refrain from categorising respondents as pertaining to one group only, as views were not discrete, but rather part of a continuum. We organised our three findings sections to reflect each one’s alignment with a mode of coordination (Moreira, 2012). In the process of doing so, we aim to provide links between the IVF add-on debate and larger healthcare values espoused by professionals.

### The market: Efficiency and innovation

The pace of innovation in fertility care is rapid. Practice in the field is significantly more advanced than it was even a decade ago. However, a majority of patients still walk away from treatment unsuccessful, while many are diagnosed with unexplained fertility. Thus, the demand for improving LBRs is high. Nonetheless, the best avenue to do so is unclear. We argue that the market creates constant conflict with the laboratory, with stakeholders deeming one as more worthy, yet not completely ruling out the other. This shows that some degree of private innovation is required to keep this field moving forward, thus making it difficult for stakeholders to completely disregard commercialisation as a potential positive force, especially when public funding for fertility care is scarce. For example, a medical director, argued that a strict EBM approach is unrealistic based on how the field has progressed:

If anybody says we always need evidence to back up what we’re doing you could argue that we’d never get off the ground. What, in my view, what we need is for people to be able to have, with good rational arguments, the opportunity to introduce something new. Now if that can be funded, supported by good research and clinical practice to provide the evidence at the outset, well that would be the perfect world, but it never happens that way. (Medical director #1, private clinic)

Similar to the respondent above, those who have been involved one way or another with clinical trials on the ground were sceptical that it is reasonable to wait until multi-site trials finish collecting data on technologies that are perceived as innovative new treatments. Talking about ICSI – a procedure now widely used as a solution for male infertility - the same respondent went further and challenged the assumption that everything needs an RCT:

For example, they [the HFEA] may have had ICSI as a red in 1993 if they were doing it then which they weren’t, right, and now it’s mainstream but it would have been a red then. And it’s never been randomised, it’s just been adopted because actually it’s the parachute story: nobody is going to do a randomised control trial on whether a parachute works. And that’s where ICSI is really, it is the only way to achieve fertilisation in some cases, so you can’t put anything but a green on it because it works but there’s been no RCT. (Medical director #1, private clinic)

Proponents of a market-based approach to IVF treatment saw themselves as more realistic than strong EBM promoters. They felt that, overall, it is just too challenging in IVF to fully adhere to strict EBM tenets. This is not to say they did not value evidence. However, EBM discourses, as deployed by the HFEA, were seen as excessively constricting optimal practice and innovation.

In addition to innovation and productivity, the market as a mode of coordination justifies the use of new technologies by aligning the practice with the ideal of freedom of choice (Moreira, 2012). Our data reveal that its proponents emphasise patient choice, especially in the context where many pay for their IVF treatment, whether this happens in private clinics or NHS clinics due to ineligibility for state funding. Although many clinicians would like to see the NHS fund all IVF cycles, some feel that, in the current environment, they have the responsibility to offer patients new options, if they become available. For example, one NHS-affiliated embryologist said that add-ons are ‘needed to make the technology available’ and that she ‘would rather the patient had to pay extra but actually had access to technology than that they didn’t have access’. Withholding treatments from patients was even seen as unethical by some. A clinician summarised their dilemma and leaned towards offering choice:

Be honest with your patients, tell them this is a new technique, tell them you think it might work but there’s no real evidence that it does work, and then let them go away and make their decision. Of course the critics of that would say well, actually you’re offering hope and as soon as you offer hope they’re going to go for it, you know. But then the flipside of that argument is what if it actually does help? And what if then you’re not offering it to the patient because you’ve not got your huge multi-centre trial with a million people in each study arm but you disadvantage them by not offering it. So there are a lot of factors to balance there. (Clinician/Professional body representative #2)

While evidence is seen as important, the respondent is still concerned with offering patients choice and access to the market. Professionals may fear a disadvantage in not offering options.

While some clinicians would encourage stricter regulation, others are more comfortable with potentially introducing new treatments quicker in the hopes that it will benefit patients struggling to conceive. Differences in views were not always demarcated according to private or public affiliation. Our respondents expressed that practices surrounding add-ons are diverse and heterogenous within all clinics, NHS-affiliated or not. This challenges the idea that the IVF public sector works under more rigid innovation protocols, while the private one will sell anything to patients who are willing to pay for unproven treatments. Rather, those who emphasise innovation still work to some extent within the confines of EBM protocols, while at the same time challenging the importance of EBM in fertility care specifically. It is this value conflict that we suggest has not been captured yet in previous literatures.

### The laboratory: Evidence and effectiveness

The early emerging discourse on IVF add-ons emphasised effectiveness, with some calling for an outright ban on treatments that have not been proven to increase LBRs. Vocal critics of add-ons include the HFEA, systematic reviewers as well as prominent clinicians involved in research. We conceptualise this EBM-focussed vision of IVF care as one that prioritises the role of ‘the laboratory’. The laboratory mode of coordination stresses knowledge about treatment outcomes through the collection of rigorous clinical trial data. As such, the solution, for its advocates, is improved knowledge through RCT data collection. Such knowledge, ideally, should be available before a new treatment is introduced to patients (especially if this option comes at an additional price). Throughout this research, we also learned that public or private affiliation does not necessarily predict strong views on EBM. Rather, strict EBM proponents tend to be more involved in overseeing guidelines, research and the production of evidence. In the absence of multi-site clinical trials for many new technologies, critics call for more research. For example, one interviewee said:

So, you know, every week in the Daily Mail there’s a new phoney thing [. . .] and I get exasperated because they send me this paper, it’s a small paper, fifty couples in either arm, is this the next thing and I end up saying um, more research is needed, it needs to be properly evaluated (Clinical research professional #1)

The respondent above expressed frustration that sensationalism takes precedence over critiques of ‘unreliable’ data. EBM advocates argued for increased evidence literacy among patients and in the media. The market was indeed conceptualised by some professionals as being in opposition to ‘good science’. However, respondents also acknowledged that, to some extent, the market does drive innovation, which is highly needed in this sector. Although they sometimes did draw upon a ‘commercialisation as detrimental’ discourse, the core of the argument presented by strong EBM proponents is based on the assumption that patients should never be offered treatments that have not been validated as effective and worth the cost. Conversely, we found that many NHS-affiliated professionals were quite sympathetic to the idea of offering patients new treatments provided they do no harm. This suggests that an emphasis on respecting strict EBM tenets does not necessarily juxtapose with NHS-affiliated roles. However, evidence issues cropped up often in interviews even when not prompted, thus showing that it is something that professionals aim to take into account.

The values underlying the laboratory mode of coordination have influenced respondent views on the provision of medical care. Even those who are not avid proponents of EBM have admitted that the BBC programme has irreversibly shaped the conversation surrounding IVF, placing the lack of evidence at the forefront. An embryologist explained that although she feels reporters misrepresented professionals’ intentions, it also made them reflect more deeply about the current state of affairs in fertility care:

So the programme was like, it was contentious because I suppose the Royal College and the British Fertility Society felt that it misrepresented the sector because they said, we actually are caring, we do care about our patients, we only want what’s best for them, [. . .] but ultimately I think it was a bit of a wake-up call that was needed about where the sector is going. (Embryologist #5, NHS)

Rather than a purely commercial versus non-commercial interests’ issue, professionals such as this one also problematised how their relationship with patients was portrayed in simplistic ways. The quote above also illustrates a tension between a science-first versus patient-first approach. This, we argue, is another key tension between different orders of worth that extends beyond private/public divides in healthcare. We further analyse this tension in the third section of our findings, where we highlight the forum mode of coordination.

The laboratory mode underlines debates about the quality of effectiveness data available. Add-on critics dislike the technological ‘hype’ and ensuing disappointment that characterises fertility care. They stress that technologies should not be adopted solely on the basis of small or retrospective studies. The gold standard of evidence (RCTs and meta-analyses of RCTs) is deemed as the only one that can determine the worth of a new treatment. For example, Cochrane review authors (Armstrong et al., 2015: 35) commented in a journal rebuttal against critics that they ‘remain convinced that randomised controlled trials must take place’ and ‘we must look to evidence-based medicine to adequately assess the safety and effectiveness (including cost-effectiveness) of these [add-on] technologies’. EBM proponents critique studies that do not adhere to strict methodological protocols, arguing that anything less than RCTs will not help clinicians in their decisions.

### The forum: Patient involvement

Concerns about patients’ welfare and their access to new treatments featured prominently in our data. This shows that professional views do take into account what they believe works for patients. Because we focus on data from professionals, we do not make inferences about the influence of patient groups. However, we here aim to demonstrate that the patient involvement mode of coordination does strongly shape clinician views and values on how IVF innovations should be introduced. The discourse of increased regulation itself is often deployed to protect what stakeholders see as ‘vulnerable patients’. Respondents that favoured a stricter EBM approach feared the gullibility of fertility patients who might try new treatments without being informed of their shortcomings. However, our study data suggests that the discourse of the ‘vulnerable patient’ that will take up any treatments in order to get pregnant is challenged in one-on-one interviews with professionals. A significant number of respondents were sceptical of the view that patients would have issues making informed decisions, if given the right information. They also stressed that some add-ons can improve quality of care, regardless of outcomes. For example, time-lapse, a tool that monitors embryos constantly, was praised for its ability to give patients additional information about their embryos and specific fertilisation issues.

Clinicians problematised the notion that all add-ons are equally non-beneficial to patients. Consequently, they challenged the use of the catch-all term of ‘add-ons’ itself because it includes lab tools such as time-lapse along with controversial treatments such as reproductive immunotherapy. While collecting our data, we observed a variety of practices with regards to payment for add-ons. Time-lapse, embryo glue and the endometrial scratch were included in some standard IVF packages offered at both NHS and private clinics. As such, it is unclear if these treatments retain the status of ‘add-on’. Some clinicians felt that if no harm is involved, technologies are not necessarily a type of ‘problematic’ add-on. In such discourses, the patient and their experience becomes a central concern for professionals. If informed patients want to access new treatments, some professionals are happy to provide these. HFEA (2020) data show that many patients are indeed interested in such treatments. In fact, one of the biggest criticisms of the strict EBM approach was its seeming ignorance of the immediate needs of fertility patients (Balen et al., 2016). Clinicians and nurses especially placed patient involvement at the top of their concerns, thus invoking the forum mode of coordination. Again, we do not suggest that evidence was not valued. Rather, it was not the top concern of those interacting with patients on a daily basis. This signals a disconnect between hard-line EBM proponents and practitioners who see the role of evidence as more ambivalent or sometimes even in conflict with a patient-centred approach. Again, this is a key source of conflict between modes that, we argue, is a result of complexity and fragmentation, rather than commercialisation alone. According to some interviewees, a top-down restrictive approach to the introduction of new treatments does not leave enough room for patients to get involved. Feedback from patients was seen as an important part of the process, as this clinician stressed:

I think that having more feedback from patients is probably the best way because, you know, you can’t be in every consultation but you can take feedback from patients about how informed they felt and whether they were told about things. And I think that’s probably the most effective way. You know, you can’t force people to be saying certain things in a consultation. (Fertility consultant #1, NHS)

Given the ever-changing fertility care landscape and the scarce public funding, some strongly felt that patients must be continually involved in decision-making processes. According to such stakeholders, a strict EBM approach would be difficult to implement in a medical field like IVF where: (1) there is a mix of public and private clinics; (2) the prices and availability of add-ons are inconsistent; (3) patients require immediate treatment; (4) there is a precedent for introducing successful treatments without RCTs to test for effectiveness. Respondents stressed an inherent tension between putting patients first and waiting for clinical trial data for new treatments.

Clinicians (many but not all) had a more lenient definition of what counts as evidence, when compared to regulators and systematic reviewers who tend to dismiss studies that are not RCTs. This signals similar values, but a different mode of coordination for immediate priorities. A nurse commented on the important role that patients have in fertility care and what she considers to be sufficient evidence:

The patients now are a very, very well educated and informed group. [. . .] So your add-ons that patients do discuss as well will be, you know, the more sort of controversial things like intralipids, PGS testing, [. . .] patients are very well informed now and they come through with a lot of things. Normally the Daily Mail or the Mail on Sunday that they bring that they’ve read an article in and want to ask about it. So I make it very clear in my practice that I will only support these things if they are clinically-based, evidence-based. So things like intralipids I won’t use. But obviously if there’s evidence suggesting they’re good things to be used and patients understand what they’re agreeing to, so yes, I will be discussing with patients. (Senior fertility nurse #3, NHS)

The respondent felt that some evidence is better than no evidence (HFEA amber versus red treatments) and stressed this in her approach. Intralipids, an intravenous infusion of fatty acids meant to reduce the body’s negative/expulsive response to a foetus, was considered more controversial to recommend to patients than other add-ons. Also building on the idea of lay agency, another professional emphasised cultural expectations in the UK healthcare system:

And this is very different, this is also very cultural, is in the UK patients are given a lot of choice. If you go back to [X] from where I come, then patients do not expect to be given choice, they have never had the option, or rather they trust what the doctor says. (Fertility consultant #2, NHS)

The imperative of patient involvement beyond the issue of adhering strictly to EBM needs to be taken into account. We observed that the desire for maintaining a good relationship with patients sometimes shapes values and decisions differently. Unsurprisingly, there is some overlap between choice as dictated by the market and choice as dictated by the forum. Nonetheless, we found that proponents of the market were more concerned with the development of new treatments, whereas advocates of the forum took pride in practicing patient-centred care. Moreover, with the advent of personalised medicine, more IVF clinicians are now steering clear from making catch-all recommendations to patients. Rather, they would prefer to see an approach where the right technologies are offered to the right patients – individuals who might benefit from specific treatments, given their particular situation. As the medical literature debate has been so focussed on the lack of evidence, it is not clear how a more personalised approach can be achieved.

## Discussion

In this article, we analysed diverging stakeholder views in the IVF evidence debate through the use of a sociological perspective on healthcare modes of coordination (Moreira, 2012), showing the alignment between these modes and different professional views. Firstly, we challenge an over-simplified view that issues surrounding innovation in fertility care can be attributed solely to public/private care divides, as has been suggested in the medical literature critiquing add-ons (Harper et al., 2017; Repping, 2019; Wilkinson et al., 2019). We argue that rather than valuing or devaluing EBM based on the status of their clinic (private or public), stakeholders have different priorities depending on experience and the coordination mode salient for them. Differences in professional approaches to innovation and the introduction of new treatments have seldom been acknowledged in the add-on debate and the scholarly literature, more widely. We contend that stakeholder views should be conceptualised in alignment with key values in contemporary medicine – values that, ultimately, are all constitutive of modern healthcare, but which have not been integrated effectively. We suggest that accounting for wider shifts in modern medicine would be beneficial in investigating healthcare sectors where evidence for innovations is uncertain. This echoes other scholars’ (Armstrong, 2002; Greenhalgh, 2014; Rabeharisoa and Bourret, 2009) call for practice and policy to account for a more complex and critical understanding of the role that EBM plays in healthcare.

Our sociological reframing of the add-on debate relates this issue to the wider IVF literature (Nahman, 2016; Pavone and Arias, 2012; Thompson, 2016; Van de Wiel, 2019; Vertommen, 2017). In this article, we show how the field of fertility care as a whole can be seen as representative of larger healthcare shifts. However, we detail how the status of EBM requires greater attention from IVF scholars due to the specificities of fertility care. As such, we stress the need to relate fertility care practices and innovation issues to the critical EBM literature (Concato et al., 2000; Deaton and Cartwright, 2018; Upshur, 2005). We contend that this is necessary for a deeper understanding of the logics of fertility care provision. On one hand, EBM has standardised tenets on the quality of evidence that is required for optimal clinical decisions (Goldenberg, 2006; Lambert, 2006), where meta-analyses of randomised controlled trials (RCTs) and RCTs are ranked as top quality (Murad et al., 2016). On the other hand, there are established medical practices that have never actually met EBM’s ‘gold standard’ of evidence (Morreim, 2003), with fertility care being a prime example of this (Perrotta and Geampana, 2021). Researchers who have studied clinical enactment of EBM have noted that, in many fields, professional knowledge and skills are still of utmost importance (Berg and Timmermans, 2000; Tonelli, 2006). In this article, we stress how this is also the case in fertility care. In addition, we suggest that more attention should be paid to cases where the use and application of EBM tenets is difficult in order to understand EBM’s systemic limitations. Our case suggest that such discussions are especially lacking in professional arenas, as many of our respondents did not feel it was appropriate to raise concerns in their circles about the potential limitations of strict adherence to EBM.

As mentioned earlier, our data only speak to how professionals conceptualise the role of patients in care and how they see themselves as taking lay choices into account in clinical decision-making. While we cannot speak about patient views, there is a vast literature (Greil et al., 2009) that has already studied patient experiences and activism. Therefore, we know that fertility care is a field where patients have been vocal. There is some indication, however, that those who are vocal tend to be those who have experienced more failed cycles and are affluent enough to be able to pay for repeated IVF cycles (Lee, 2017). Patient advocacy strategies also vary across cultures (Culley et al., 2012; Gunnarsson Payne and Korolczuk, 2016). While reviewing media and medical documents, we have not seen anything to suggest that patients might have a unified view on add-ons. Professional accounts and previously published work (Perrotta and Hamper, 2021) suggest that patients can have divergent views on add-ons, with some more open to them than others. Nonetheless, the salient point that we make in this article is that professionals are inclined to question strict EBM tenets if they feel a new treatment might benefit patients and patients are informed about the lack of evidence. As such, we make a connection between the laboratory and forum modes of coordination to show that, depending on how they conceptualise patients’ rights to make informed choices, professionals will form a certain view on whether or not unproven treatments need to be withheld at all costs. Thus, we reinforce the view that patient-centeredness and choice are underlined by trust and communication between patients and carers (Mol, 2008). Nonetheless, informed choice might not be easily achieved in practice. For example, patients are not always content with the information they are provided (Mounce, 2013). In addition, the time-sensitive nature of infertility treatment might also make choices difficult (Culley et al., 2012). Consequently, how the forum mode of coordination is achieved in clinical practice requires further investigation.

The use of Moreira’s (2012) framework has allowed us to make links between different aspects of fertility care and show how, in some sectors, modern healthcare values co-exist in tension, without a clear resolve. We suggest that a key aspect of fertility care in the UK is the blurry line between private and public care, where similar practices and views can be seen in both. As we have outlined elsewhere (Geampana and Perrotta, 2021; Perrotta and Geampana, 2020), NHS clinics feel drawn into similar practices to private clinics. The market logic seeps into the healthcare sector as a whole, making it harder to delineate the two arenas: a phenomenon observed by others (Conrad, 2005; Greene, 2007; Light, 2010; Timmermans and Oh, 2010) as well. It can be said that the competitiveness of the sector drives technological change, but we argue that a public/private divide is not what defines different attitudes towards the evidence behind new technologies. We suggest that the analytical value of the modes of coordination framework could be improved if it can be refined to better anticipate where tensions and conflict can occur or, rather, in which fields/cases it is likely that the three modes will not coexist peacefully. We further suggest that conflict might arise when a sector with a mix of public and private elements has a strong drive for technological innovation in a context where EBM is highly valued (here, the UK). The latter is arguably a feature of the NHS as a whole, therefore attention needs to be paid to how a strong EBM orientation conflicts with patients’ willingness to try new treatments if they wish to do so. Evidence debates can become more contentious within UK health sectors due to the increasing complexity of privatisation initiatives and the lack of clear responsibility on which organisations have to collect data and produce evidence for new treatments. To understand innovation debates, however, we contend it is useful to look at the values upon which healthcare priorities are set.
